# Developing an Outcome Measures in Rheumatology (OMERACT) Core set of Outcome Measures for FOot and ankle disorders in RheumaTic and musculoskeletal diseases (COMFORT): core domain set study protocol

**DOI:** 10.1186/s13063-023-07104-7

**Published:** 2023-01-28

**Authors:** Lara S. Chapman, Anthony C. Redmond, Caroline A. Flurey, Pamela Richards, Toby O. Smith, John B. Arnold, Dorcas Beaton, Philip G. Conaghan, Yvonne M. Golightly, Marian T. Hannan, Catherine Hofstetter, Lara J. Maxwell, Hylton B. Menz, Beverley Shea, Peter Tugwell, Philip Helliwell, Heidi J. Siddle

**Affiliations:** 1 Leeds Institute of Rheumatic and Musculoskeletal Medicine, University of Leeds, Leeds, UK; 2 Centre for Sport, Exercise and Osteoarthritis Research Versus Arthritis, Nottingham, UK; 3 National Institute for Health Research (NIHR) Leeds Biomedical Research Centre, Leeds, UK; 4 Department of Health and Social Sciences, Faculty of Health and Applied Sciences, University of the West of England, Bristol, UK; 5 OMERACT Patient Research Partner, Bristol, UK; 6 Warwick Clinical Trials Unit, University of Warwick, Warwick, UK; 7 IIMPACT in Health, Allied Health & Human Performance Unit, University of South Australia, Adelaide, Australia; 8 Institute for Work and Health, and Department of Occupational Science and Occupational Therapy, Rehabilitation Sciences Institute, University of Toronto, Toronto, ON Canada; 9 Thurston Arthritis Research Center, University of North Carolina at Chapel Hill, Chapel Hill, NC USA; 10 College of Allied Health Professions, University of Nebraska Medical Center, Omaha, NE USA; 11 Hinda and Arthur Marcus Institute for Aging Research, Hebrew SeniorLife, Beth Israel Deaconess Medical Center, Boston, MA USA; 12 Harvard Medical School, Boston, MA USA; 13 OMERACT Patient Research Partner, Toronto, ON Canada; 14 Faculty of Medicine, University of Ottawa, Ottawa, ON Canada; 15 School of Allied Health, Human Services and Sport, La Trobe University, Melbourne, VIC Australia; 16 Clinical Epidemiology Program, Ottawa Hospital Research Institute, Ottawa, ON Canada; 17 Department of Medicine, University of Ottawa, Ottawa, ON Canada

**Keywords:** Foot, Ankle, Rheumatology, Musculoskeletal, Core outcome set, Outcome measures, Consensus

## Abstract

**Background:**

Foot and ankle involvement is common in rheumatic and musculoskeletal diseases (RMDs). High-quality evidence is lacking to determine the effectiveness of treatments for these disorders. Heterogeneity in the outcomes used across clinical trials and observational studies hinders the ability to compare findings, and some outcomes are not always meaningful to patients and end-users. The Core set of Outcome Measures for FOot and ankle disorders in RheumaTic and musculoskeletal diseases (COMFORT) study aims to develop a core outcome set (COS) for use in all trials of interventions for foot and ankle disorders in RMDs. This protocol addresses core outcome domains (*what* to measure) only. Future work will focus on core outcome measurement instruments (*how* to measure).

**Methods:**

COMFORT: Core Domain Set is a mixed-methods study involving the following: (i) identification of important outcome domains through literature reviews, qualitative interviews and focus groups with patients and (ii) prioritisation of domains through an online, modified Delphi consensus study and subsequent consensus meeting with representation from all stakeholder groups. Findings will be disseminated widely to enhance uptake.

**Conclusions:**

This protocol details the development process and methodology to identify and prioritise domains for a COS in the novel area of foot and ankle disorders in RMDs. Future use of this standardised set of outcome domains, developed with all key stakeholders, will help address issues with outcome variability. This will facilitate comparing and combining study findings, thus improving the evidence base for treatments of these conditions. Future work will identify suitable outcome measurement instruments for each of the core domains.

**Trial registration:**

This study is registered with the Core Outcome Measures in Effectiveness Trials (COMET) database, as of June 2022: https://www.comet-initiative.org/Studies/Details/2081

## Background and objectives

Rheumatic and musculoskeletal diseases (RMDs) significantly impact on the economy and healthcare resources, and their prevalence is projected to increase [1]. Foot and ankle disorders are common in RMDs. Around 90% of people with rheumatoid arthritis experience foot and ankle problems, including pain, rearfoot and forefoot deformity and peripheral arthritis [2, 3]. In spondyloarthropathies, foot problems also include dactylitis and enthesitis; in psoriatic arthritis specifically, forefoot deformity affects over 90% of people and two thirds experience foot pain [3]. Foot involvement is a cardinal feature of gout [4], and foot and ankle osteoarthritis affects almost 17% of people aged 50 and over [5]. Foot complaints are also prevalent in systematic lupus erythematous [6] and systemic sclerosis [7]. Foot and ankle disorders in RMDs are associated with significant disability, affecting tasks essential to daily living, and leading to loss of independence and reduced quality of life [8–10].

Foot and ankle disorders in RMDs have gained attention in recent years. There has been an increase in the number of clinical trials and observational studies investigating interventions for these conditions. Pharmacological (e.g. local and systemic drug therapies), conservative (e.g. footwear and foot orthoses) and surgical interventions have been shown to reduce foot and ankle symptoms in people with RMDs [11–18]. Despite widespread use of these treatments in clinical practice, there remains a lack of high-quality evidence assessing their effectiveness [19]. Furthermore, translation of existing research evidence into practice is generally poor [19, 20]. A significant factor contributing to these issues is the heterogeneity of outcome measures used across trials, hindering the ability to compare findings between trials and to pool data in meta-analyses [21–25]. Additionally, it is widely agreed that if research findings are to influence policy and practice, trial outcomes need to be important, relevant and feasible for all key stakeholders, especially patients and health professionals [26–28]. Previous studies have shown that outcome domain priorities in RMDs differ between patients and health professionals [29–31]. In foot and ankle disorders specifically, it is suggested that clinicians consistently underestimate the psychosocial impact of these conditions [32, 33] and discrepancies have been identified between participants’ presenting concerns and the treatments suggested by clinicians.

One solution to reduce outcome heterogeneity and improve transferability of trial findings into practice is to develop a core outcome set (COS). This is an agreed, standardised set of outcomes that is measured and reported, as a minimum, in all clinical trials in a specific area of health [28]. A key factor when developing a COS is the involvement of all key stakeholders, to ensure clinically relevant outcomes are identified. A COS consists of a core domain set and core outcome measurement set; core domain sets provide guidance on *what* outcomes to measure in all trials of a specific condition, whilst core outcome measurement sets provide guidance on *how* to measure different outcomes [34]. COSs have been developed for various health conditions and areas of the body to reduce variation in outcome measurement in clinical trials [35–38]. There are currently no published COSs for foot and ankle disorders in RMDs, and no existing COSs for RMDs have included outcomes relating to the foot and ankle. A recent protocol has detailed the development of an international core domain set for ankle OA, but this does not include the foot or consider any other RMDs in which foot and ankle problems are common [39].

The Outcome Measures in Rheumatology (OMERACT) Initiative was established in 1992 and has been successful in improving outcome measurement for many RMDs and areas of the body, through the development of COSs, and increased consistency in outcome measurement has been observed after dissemination of OMERACT COSs [40, 41]. In 2018, an international group, the OMERACT Foot and Ankle Working Group, comprising patients, clinicians and researchers, convened to start to develop an internationally agreed COS for foot and ankle disorders in RMDs. It is anticipated that this COS will increase consistency in outcome measurement for research investigating the effectiveness or efficacy of interventions for foot and ankle disorders in RMDs, leading to improved evidence on treatments for patients, with a potential subsequent reduction in burden on health services. This protocol focuses on developing a core *domain* set for the COS and outlines the methods to achieve this.

The objectives of the *C*ore set of *O*utcome *M*easures for *FO*ot and ankle disorders in *R*heuma*T*ic and musculoskeletal diseases (COMFORT): Core Domain Set study are to:

(i) Establish the frequency and scope of outcome domains previously reported in clinical trials of interventions for foot and ankle disorders in RMDs;
(ii) Identify additional outcome domains of importance to patients with RMDs who have experienced foot and ankle disorders;
(iii) Achieve multidisciplinary, multi-stakeholder and expert international consensus and endorsement of a core set of outcome domains for foot and ankle disorders in RMDs.

## Scope

The COS will apply to measuring the efficacy or effectiveness of pharmacological, conservative and surgical interventions in randomised controlled trials (RCTs), controlled clinical trials and observational studies for patients with RMDs and foot and ankle disorders. For this core domain set, RMDs encompass inflammatory arthritis, osteoarthritis, spondyloarthropathies, connective tissue diseases, crystal arthropathies and musculoskeletal disorders (e.g. plantar heel pain and tendinopathies affecting the foot and ankle in the absence of systemic disease). It is anticipated that the core domain set will be relevant to patients and the public, researchers, clinicians, policymakers, guideline developers, clinical commissioners and industry representatives. The presupposition is that the core domain set will be primarily used in future clinical research. However, it will also be applicable to quality improvements and guideline development within clinical practice and could provide valuable information to healthcare professionals measuring outcomes within clinical appointments.

## Methods

### Design

COMFORT is a mixed-methods study involving literature reviews and qualitative data to inform a Delphi study and subsequent consensus meeting. The methodology for this project is adapted from the OMERACT Framework 2.1 [34, 42] and COMET Handbook [28]. Similar methods have been used in the development of other core domain sets [35, 36]. This project is registered with the COMET Initiative: https://www.comet-initiative.org/Studies/Details/2081. The reporting of this protocol adheres to the Core Outcome Set-STAndardised Protocol Items (COS-STAP) checklist [43].

### OMERACT foot and ankle working group

The COMFORT study will utilise an international group of experts to develop the core domain set. The OMERACT Foot and Ankle Working Group includes over 50 members representing Europe, Australia and North America.

Members of the working group include patients, podiatrists, rheumatologists, physiotherapists, occupational therapists, epidemiologists, psychologists, physiatrists, biomedical engineering foot and ankle researchers and industry representatives. The development of the COS is led by a steering committee, comprising four co-chairs (HJS, PH, MTH, HBM), five other contributors with expertise in the foot and ankle, RMDs and outcome measures (ACR, CAF, TOS, JBA, YMG), two patient and public involvement (PPI) contributors (PR, CH) and one fellow (LSC). The steering committee is overseen by OMERACT senior methodologists (BS, LM) and three OMERACT management team members (PC, PT, DB). Members of the steering committee communicate with each other and with the wider working group through email and teleconference. Steering committee meetings are held monthly, and all members will be involved in the design, conduct, analysis and dissemination of each phase of the research.

### Stakeholder involvement

In addition to the research team above, the following key stakeholders will be involved in developing the core domain set as participants: patients, health professionals, researchers and industry representatives. Patients with RMDs have valuable insights into the experience of living with foot and ankle disorders. With the ultimate aim of improving outcomes for patients, the patient perspective will be integrated during every phase. Patients will be invited to participate in the qualitative interviews, focus groups and Delphi study. They will be identified and recruited by working group members who have clinical roles and through patient organisations and social media campaigns.

Healthcare professionals have insight into the manifestation of symptoms, prognosis and management of patients. Healthcare professionals from different disciplines (e.g. medicine, podiatry, physiotherapy, prosthetics and orthotics, occupational therapy, orthopaedic surgery) who have clinical experience of managing patients with RMDs who present with foot and ankle disorders will be invited to participate in the Delphi study. Co-chairs and other members of the working group who work or have worked clinically will nominate suitable healthcare professionals, as well as industry (e.g. pharmaceutical, orthotic, rehabilitation medical device, assisted living technology, and orthopaedic footwear) representatives, policymakers and commissioners. These stakeholders will also be recruited through professional organisations and social media campaigns. Initial contacts who are unable to participate will be requested to nominate other similar individuals.

Researchers have insight into the feasibility of measuring outcomes in the context of clinical research. Clinical researchers known by the co-chairs of the working group to have expertise in foot and ankle disorders in RMDs will be invited to participate. Researchers will be identified and recruited by direct personalised contact, by working group members based in research institutions and at foot and ankle sessions at international meetings/relevant scientific conferences (e.g. British Society for Rheumatology (BSR), European Alliance of Associations for Rheumatology (EULAR), American College of Rheumatology (ACR) and Osteoarthritis Research Society International (OARSI)). The working group will also compile a list of researchers who have published on foot and ankle disorders in RMDs over the last 12 months, via PubMed searches, and these researchers will be invited to participate in the Delphi study. Details are provided in the relevant sections subsequently, but it is anticipated that approximately 200 contributors from a range of backgrounds will have input during the core domain set development process.

## Phase 1—identification of important outcome domains

### Phase 1a: scoping review

A scoping review will be undertaken to identify outcome domains used in existing studies investigating the efficacy or effectiveness of pharmacological, conservative and surgical interventions for foot and ankle disorders in RMDs.

#### Search strategy

The following databases will be searched from inception: Ovid Medline, Ovid Embase, Cumulative Index of Nursing and Allied Health, Cochrane Central Register of Controlled Trials and Physiotherapy Evidence Database. Additionally, the following trial registries will be searched for ongoing and future trials in this area: ClinicalTrials.gov, International Standard Randomised Controlled Trial Number registry and the Australian New Zealand Clinical Trials Registry. The review will include RCTs, controlled clinical trials, controlled before-after studies, longitudinal observational studies, cross-sectional observational studies, cohort studies and case-control studies. Published protocols and trial registry entries with clear descriptions of the intended outcome domains will also be included. Only English language studies published as full articles will be included. Individual case reports, case series, editorials and commentaries will be excluded. Relevant systematic reviews will be screened to ensure no studies meeting the scoping review inclusion criteria are missed.

#### Data extraction

Two reviewers will independently assess all studies for inclusion. Titles and abstracts will be screened, followed by full papers. Any disputes will be settled by a third independent reviewer. The following data will be extracted: study details (authors, year of publication), design, participants (sample size, type of foot and ankle disorder, type of RMD) setting (country), duration of follow-up, intervention type (pharmacological, conservative, surgical), type of intervention, comparator), outcome domains and outcome measurement instruments.

#### Analysis

Outcome domains will be classified into the core areas described in the OMERACT framework [42], comprising of three core areas—death, life impact and pathophysiological manifestations—and one strongly recommended area—resource use. Members of the steering committee will be invited to a meeting to provide feedback on the classification of outcome domains and to facilitate refinement and amalgamation of domains. Disputes will be settled by group discussion. Findings will be presented as descriptive statistics and frequency distributions.

### Phase 1b: systematic review of qualitative studies

A systematic review of qualitative studies will be undertaken to understand what outcome domains are important to patients and should be considered for inclusion in the core domain set and how these compare to domains that have been measured by researchers. This will be achieved by exploring the perceptions and experiences of people with RMDs who live with foot and ankle disorders and the impact of these disorders on their daily lives.

#### Search strategy

The following databases will be searched from inception: Ovid Medline, Ovid Embase, Cumulated Index to Nursing and Allied Health Literature and Ovid PsycINFO. Any study in which the authors have used qualitative interviewing or focus group methods to explore the perceptions and experiences of people with RMDs who live with foot and ankle disorders will be eligible for inclusion. Conference abstracts, review articles and articles not written in English will be excluded. Relevant systematic reviews will be screened to ensure no studies meeting the scoping review inclusion criteria are missed.

#### Data extraction

Two reviewers will independently assess all studies for inclusion. Titles and abstracts will be screened, followed by full papers. Any disputes will be settled by a third independent reviewer. The following data will be extracted: study details (authors, year of publication), design, participants (demographic characteristics, number, type of foot and ankle disorder, time of RMD) setting (country), data collection method (e.g. focus group, semi-structured interview), data analysis method (e.g. thematic analysis, grounded theory), approach to data analysis (e.g. inductive or deductive coding of themes) and study findings (including themes, subthemes and supporting quotes). Two authors will independently assess the quality of the included studies using the Critical Appraisal Skills Programme (CASP) Qualitative Studies Checklist (https://casp-uk.net/). A summary table detailing the presence or absence of the components of each CASP question will be produced. Any disagreements during screening or data extraction will be resolved via discussion or through inclusion of a third author. The CASP checklist does not have a scoring method and therefore a narrative summary of the quality of the individual included studies will be provided.

#### Analysis

A thematic synthesis approach will be undertaken [44]. Two reviewers will code the text ‘line-by-line’, develop ‘descriptive themes’ and generate ‘analytical themes’ to identify outcome domains of importance. The two reviewers will independently assess the confidence in the findings of the thematic synthesis using the GRADE-CERQual approach [45]. Key review findings, confidence judgements for each finding and an explanation of each judgement, will be presented in a Summary of Qualitative Findings table. Outcome domains will be added to the OMERACT framework results from phase 1a. Members of the steering committee will be invited to a meeting to provide feedback on the classification of outcome domains and to facilitate further refinement and amalgamation of domains. Findings from both reviews and a preliminary domain framework will be presented and discussed with the OMERACT Foot and Ankle Working Group and with the wider OMERACT community during OMERACT Special Interest Group (SIG) meetings in 2022 and 2023, respectively.

### Phase 1c: interviews and focus groups with patients

Primary qualitative research will be conducted to identify and understand any additional outcome domains of importance to patients with foot and ankle disorders in RMDs, explore the range and scope of domains and establish appropriate domain language and definitions.

#### Design

A phenomenological approach to qualitative research design will be employed, providing rich insight by placing emphasis on understanding the lived experiences and actions of individuals from their own points of view [46]. Qualitative research is considered a necessary step as the gold-standard for developing a core domain set and is often undertaken to inform the first round of a Delphi study, ensuring identification of meaningful outcome domains with informative and accessible descriptions [28, 34, 47]. Consolidated Criteria for Reporting Qualitative Research guidelines will be followed throughout this subphase [48]. Qualitative interviews with National Health Service (NHS) patients in the United Kingdom (UK) will be undertaken in the first instance, followed by focus groups in at least three continents, representative of relevant healthcare systems globally.

#### Participants and recruitment

Participants will be patients with RMDs who are receiving or have previously received treatment for foot and ankle disorders. Participants will be aged 18 and upwards, capturing the lived experience as well as longer term impact. Inclusion and exclusion criteria are displayed in Table 1. Based on OMERACT recommendations and previous qualitative research conducted as part of core domain set development [49–51], 15 to 20 individual interviews and at least four focus groups with five to eight participants per group will take place. A purposive sample will be recruited, based on clinical condition (as described in the Scope section above) and type of intervention (pharmacological, conservative, or surgical). The working group aims to recruit participants with a broad range of demographic (age, gender, socioeconomic status, educational level, and ethnicity) and clinical (type, duration, and severity of condition) characteristics. Interview and focus group participants will be patients identified through podiatry and rheumatology departments, through members of the working group in clinical roles and with clinical links.

**Table 1:** Participant inclusion and exclusion criteria for phase 1c (qualitative interviews and focus groups with patients)

**Inclusion criteria**	
Aged 18 or over	
Diagnosis of a RMD and have received treatment (conservative, pharmacological or surgical) for a foot and/or ankle disorder within the last 12 months	
Able to give informed consent	
**Exclusion criteria**	
Acute trauma or injury to the foot/ankle (e.g. fracture, rupture, sprain), or a sports injury	
Co-morbidities that affect the foot/ankle (e.g. diabetes, neurological conditions, including peripheral neuropathy, or peripheral arterial disease)	
Lack capacity to provide informed consent	

#### Data collection

Data will be collected through individual semi-structured interviews and focus groups. Interviews will allow participants with RMDs to share important experiences of living with foot and ankle disorders, the treatments they have received and their treatment expectations. Focus groups will allow a large amount of information to be captured across different countries in a relatively short time period and facilitate idea generation through synergistic discussion between participants [47]. Interviews will be conducted face-to-face, online (on Zoom or Microsoft Teams) or by telephone, depending on patient preference, to allow for national recruitment and to maximise inclusivity. Focus groups will be conducted online and moderated by an experienced facilitator. A topic guide for the interviews and focus groups will be developed based on previous relevant literature, input from PPI contributors, and discussions with multidisciplinary health professionals and researchers. The initial topic guide will be structured around the following topics: nature of foot/ankle problems, symptoms experienced, impact of foot/ankle problems on daily life (social; physical—including impact on general health and fitness; emotional—including impact on self-esteem; occupational), treatments received and expectations of treatments. The topic guide will be iteratively modified throughout this phase of the research. This will ensure that any areas raised by earlier participants, but not included in the initial topic guide, are covered in subsequent discussions. Interviews and focus groups will be conducted with no time limits and free discussion will be encouraged to enable thorough exploration of experiences of those with RMDs who live with foot and ankle disorders, any treatments received and treatment expectations. For interview participants whose first language is not English, interpreters will be available to aid during the interviews, promoting inclusivity. Field notes will be written during the interviews and focus groups to record non-verbal cues/contextual details. Interviews and focus groups will be digitally recorded and transcribed verbatim.

#### Data analysis

Data analysis will be undertaken concurrently with data collection, using reflexive thematic analysis to identify candidate domains and facilitate understanding of domain prioritisation amongst patients [52]. Transcript data will be managed using NVivo software (QSR International, Burlington, USA). The first phase of the analysis will involve familiarisation with the data through reading and re-reading of the transcripts by one reviewer. Initial codes will then be generated. Coding will be conducted iteratively, with new codes being added as the analysis progresses. The next phase will involve identifying and refining themes by considering how the codes are related, through discussion with Steering Committee members. Verbatim quotations will be extracted from transcripts to represent the authentic voice of participants for each theme [52]. Two experienced PPI contributors (PR, CH) will assist with interpretation of the qualitative study findings.

#### Key stakeholder feedback

A domain framework based on all findings from phase 1 will be constructed through consultation with working group members. The meaning of each domain will be discussed, and domains will be amalgamated where appropriate. A final long list of domains will be compiled, with plain English definitions.

## Phase 2—prioritisation of outcome domains

### Phase 2a: Delphi consensus study

An international, online, modified Delphi survey will be undertaken to gain consensus on the most important outcome domains to measure in future trials. The Delphi method is frequently used to achieve consensus during COS development as it allows for wide geographic dispersion and for large numbers of key stakeholders to participate, and responses are anonymous, which avoids the effect of dominant individuals [28].

#### Participants and recruitment

Patients, healthcare professionals, researchers, industry representatives, guideline developers, policymakers and clinical commissioners will be recruited, as detailed in the stakeholder involvement section above. There is no consensus on the optimal sample size for a Delphi study [28]; however, the working group will endeavour to start with 100 participants in each stakeholder group. The final sample size will be determined by timeframe, but based on previous OMERACT Delphi studies, is expected to be between 150 and 200 participants [34]. The first round of the survey will be open for 6 weeks, and each subsequent round, 3 weeks. A reminder will be sent 3 weeks after the initial invitation to the first round, and 1 week after invitations to subsequent rounds. Four rounds will take place.

The Delphi study will be promoted through working group members’ academic and clinical networks, recruitment information flyers in clinical settings, PPI events, relevant conferences and meetings, social media and patient and professional organisations. Potential participants indicating interest in taking part in the Delphi will be provided with information regarding its purpose, what is expected of them (including expectations about intended time commitments), the long-term benefits of participation, and the importance of completing all rounds. In addition to a written information sheet, a link to a video developed with PPI contributors will be sent to potential participants. This will explain the concept of the Delphi study and the wider context of core outcome sets in plain language. Participants will complete an online electronic consent form and a short demographic questionnaire prior to the first round of the Delphi. This will include a ‘tick box’ for expressions of interest for attendance at a subsequent consensus meeting. The initial survey and background information will be reviewed by the working group and pilot tested with steering committee members.

#### Data collection and analysis

Data will be collected using online surveys via DelphiManager. The first round of the survey, generated from the findings of phase 1 of the COMFORT study, will be presented to participants. Participants will be asked to rate on a scale of one to nine the importance of each outcome domain to be included in the core outcome set. Scores of one to three will correspond to not important, four to six to important but not a priority and seven to nine to very important and a priority. Participants will also be able to suggest additional domains, amalgamation of domains, wording/definition modifications and make any other comments in free text. Participants’ feedback, response rates and results will be recorded; data will be analysed using IBM SPSS® Statistics software (IBM Corporation, Armonk, NY, USA) and presented as descriptive statistics and frequency distributions. Content analysis will be used to organise free text responses and elicit meaning from the data, and the results will be used to revise and develop domains for inclusion in subsequent rounds. An individual’s own score and the responses of each stakeholder group will be fed back to participants after each round, allowing them to consider the views of others before re-rating each domain. Any new outcome domains suggested will be reviewed by the Working Group Steering Committee and discussed for inclusion in the next round.

Following established OMERACT guidelines, data will be analysed by two groups: patients and other stakeholders. After each round, any domains where ≥ 70% of participants in both stakeholder groups voted the domain as ‘not important’ (score one to three) will be excluded from the Delphi list. All the remaining domains and any new domains approved for inclusion will then be re-scored in the next round. To aid retention, personalised reminder emails will be sent to participants, in addition to prompts and updates from working group members on social media. Non-responders/partial responders will be excluded from subsequent rounds. At the end of round 3, a preliminary core set of outcome domains will be determined, consisting of those scored as ‘critically important’ (scores seven to nine) by both stakeholder groups. Outcome domains that are scored as ‘critically important’ by ≥ 70% of participants in one of the stakeholder groups (patients *OR* other stakeholders) will be considered ‘important-but-optional’ outcome domains. A final Delphi ‘sorting round’ will take place, during which participants will be asked which domains they feel are important to keep *in* or *out* of the core domain set.

### Phase 2b: consensus workshop

The preliminary core set of outcome domains and their definitions will be presented and discussed at an international consensus workshop hosted by OMERACT. A virtual workshop is expected to take place in 2024, permitting participants to join remotely to facilitate wider international input. Based on previous OMERACT consensus workshops [35, 36], the minimum target number of participants for the consensus workshop will be 100. Participants who completed all rounds of the Delphi and who expressed interest in participating in the consensus meeting will be invited to attend, in addition to wider members of the multidisciplinary, multi-stakeholder OMERACT community. Participants will be sent a copy of the results from phases 1 and 2 prior to the workshop and asked to consider the results to date so that they are able to give informed feedback in the meeting. This pre-reading will be accompanied by a video summarising the working group’s findings to date. All parts of the workshop will be audio-recorded and transcribed. The preliminary format for the consensus meeting is as follows:

#### Part 1: plenary introduction

The aims, methods and findings from phases 1 and 2 will be presented by the OMERACT Foot and Ankle Working Group co-chairs, PPI contributors and fellow.

#### Part 2: breakout groups

Participants will be assigned to breakout groups, with representation from each different stakeholder group in attendance, including patients, to facilitate discussion from different perspectives. Breakout groups will be led by a facilitator and co-facilitator from the steering committee. The facilitators will encourage active discussion from all attendees, moderate the discussion and take notes.

#### Part 3: plenary discussion and final vote

Participants will then reconvene in the plenary session, moderated by an OMERACT Foot and Ankle Working Group co-chair. Each breakout group facilitator will report the outcome of their respective breakout discussions to the wider group, and further discussion will be encouraged. If there is any disagreement among stakeholders at this stage, qualitative findings from phase 1b and 1c will be used to inform further discussion and facilitate resolution [47]. The final core set of outcome domains will be determined by an anonymous electronic vote on each proposed outcome domain. All outcome domains voted for by ≥ 70% of participants will be included in the core set.

### Ethical approval/informed consent

Ethical approval for has been granted from the North East - Tyne and Wear South Research Ethics Committee and the Health Research Authority for Phase 1c of this study (reference 22/NE/0226). Informed consent will be sought from COMFORT participants prior to participation, using paper or electronic consent forms. The COMFORT study will be conducted according to the Declaration of Helsinki.

### Dissemination

A strategy to promote uptake of the core domain set will be finalised with the OMERACT Foot and Ankle Working Group. Findings from each phase of the research will be published in relevant, peer-reviewed academic journals and on the OMERACT and COMET websites, where the project is registered. Members of the OMERACT Foot and Ankle Working Group Steering Committee will disseminate the research through social media and at professional conferences, across disciplines of foot/ankle (e.g. UK Royal College of Podiatry, Australian Podiatry Association), rheumatology (e.g. BSR, ACR, EULAR) and outcome measurement (e.g. COMET, Patient Reported Outcome Measures Annual Research conference) and to rheumatology and musculoskeletal clinical teams. The research team will also work with Cochrane review groups and journal editors to disseminate findings and increase uptake, ask funding bodies to consider including the core domain set in applications for financial support for future studies and recommend that guideline developers include the core domain set in recommendations on the management of patients with RMDs and foot and ankle disorders. Finally, the research team will continue to engage with PPI contributors to work with the committees and communities they are involved with to promote core domain set uptake, and findings will be disseminated through newsletters and articles to charities and patient organisations. Future uptake of the core domain set will be measured by citation tracking.

## Discussion

This protocol has outlined the methodology that will be used to establish the first internationally agreed, standardised core set of outcome domains to be measured and reported, as a minimum, in future clinical trials and observational studies of foot and ankle disorders in RMD. The core domain set will contribute to a future COS, which will incorporate core domains and core outcome measurement instruments.

A key strength of this study is its international involvement from a wide range of key stakeholders, including patients and multidisciplinary health professionals, ensuring that clinically meaningful outcome domains will be identified. Additionally, the active involvement of international clinical trials experts throughout the core domain set development process will facilitate uptake. A limitation of this research is that it currently lacks input from low- and middle-income countries, and the Delphi is restricted to the English language and those with internet access. However, interpreters will be available during the qualitative phase of this research to permit inclusion of patients whose first language is not English, in an effort to recruit participants from under-represented groups.

Following development of this core domain set, additional work will be undertaken to identify outcome measurement instruments for the core domains. A critical appraisal and synthesis of the psychometric evidence for outcome measurement instruments used in clinical trials and observational studies of interventions for patients with all foot and ankle disorders in RMDs will be undertaken, alongside a feasibility evaluation, using established guidelines [53, 54]. Any gaps in the available instruments for the core domains will be identified and addressed through further work.

## Conclusions

If implemented successfully, the core domain set should improve research efficiency and support comparison and combination of results across different trials, thus improving the quality of evidence for foot and ankle treatments. Additionally, it will facilitate translation of research findings to clinical practice, ensuring that these findings lead to improved clinical care.

## Trial status

The scoping review (phase 1a) and systematic review (phase 1b) have recently been completed. The results were presented in a virtual OMERACT SIG in October 2022. Recruitment to patient interviews and focus groups (phase 1c) is anticipated to commence in January 2023.

## Data Availability

Not applicable.

## Abbreviations

ACR: American College of Rheumatology
BSR: British Society for Rheumatology
CASP: Critical Appraisal Skills Programme
COMET: Core Outcome Measures in Effectiveness Trials
COMFORT: Core set of Outcome Measures for FOot and ankle disorders in RheumaTic and musculoskeletal diseases
COS: Core outcome set
EULAR: European Alliance of Associations for Rheumatology
NHS: National Health Service
OMERACT: Outcome Measures in Rheumatology
PPI: Patient and public involvement
RCT: Randomised controlled trial
RMD: Rheumatic and musculoskeletal disease
OARSI: Osteoarthritis Research Society International
UK: United Kingdom
